# Ethyl Ferulate, a Component with Anti-Inflammatory Properties for Emulsion-Based Creams

**DOI:** 10.3390/molecules19068124

**Published:** 2014-06-17

**Authors:** Ana C. Nazaré, Carolina M. Q. G. de Faria, Bruna G. Chiari, Maicon S. Petrônio, Luis O. Regasini, Dulce H. S. Silva, Marcos A. Corrêa, Vera L. B. Isaac, Luiz M. da Fonseca, Valdecir F. Ximenes

**Affiliations:** 1 Departamento de Análises Clínicas, Faculdade de Ciências Farmacêuticas de Araraquara, UNESP, Universidade Estadual Paulista, Araraquara-SP, 14801-902, Brazil; 2 Departamento de Cosmetologia, Faculdade de Ciências Farmacêuticas de Araraquara, UNESP, Universidade Estadual Paulista, Araraquara-SP, 14801-902, Brazil; 3 NuBBE - Núcleo de Bioensaios, Biossíntese e Ecofisiologia de Produtos Naturais, Departamento de Química Orgânica, Instituto de Química, UNESP, Universidade Estadual Paulista, Araraquara-SP, 14800-900, Brazil; 4 Departamento de Química, Faculdade de Ciências, UNESP, Universidade Estadual Paulista, Bauru-SP, 17033-360, Brazil

**Keywords:** neutrophil, NADPH oxidase, oxidative stress, ferulic acid, ethyl ferulate, cosmetic product

## Abstract

Ethyl ferulate (FAEE) has been widely studied due to its beneficial heath properties and, when incorporated in creams, shows a high sun protection capacity. Here we aimed to compare FAEE and its precursor, ferulic acid (FA), as free radical scavengers, inhibitors of oxidants produced by leukocytes and the alterations in rheological properties when incorporated in emulsion based creams. The cell-free antiradical capacity of FAEE was decreased compared to FA. However, FAEE was more effective regarding the scavenging of reactive oxygen species produced by activated leukocytes. Stress and frequency sweep tests showed that the formulations are more elastic than viscous. The viscoelastic features of the formulations were confirmed in the creep and recovery assay and showed that the FAEE formulation was less susceptive to deformation. Liberation experiments showed that the rate of FAEE release from the emulsion was slower compared to FA. In conclusion, FAEE is more effective than FA as a potential inhibitor of oxidative damage produced by oxidants generated by leukocytes. The rheological alterations caused by the addition of FAEE are indicative of lower spreadability, which could be useful for formulations used in restricted areas of the skin.

## 1. Introduction

Ferulic acid (4-hydroxy-3-methoxycinnamic acid, FA) is a naturally occurring plant product that has long been studied due to its potential beneficial health properties, including anti-oxidative [1], anti-inflammatory [2,3,4], neuroprotective [5,6] and antiproliferative activities [7]. The efficiency of FA as an antioxidant compound has been linked to the stability of its phenoxyl radical due to charge delocalization between the aromatic ring and the double bond in the side chain [8].

In the same direction, the alkyl ester derivative of ferulic acid, ethyl ferulate (ethyl 4-hydroxy-3-methoxycinnamate, FAEE) has also been widely studied and some recent findings include its anticholinesterase activity [9]; inhibition of nuclear factor-kappa B (NF-κB) activity in LPS-stimulated RAW 264.7 macrophages [10]; inhibition of inducible nitric oxide synthase (iNOS) induction in UV-induced oxidative stress in melanocytes [11]; induction of heme oxygenase (HO-1) activity in rat astrocytes and neurons, which is a putative pathway against oxidative stress that underline neurodegenerative diseases [12]; and cytoprotective effect against reactive oxygen species (ROS)-induced damage through the stimulation of dermal fibroblasts stress response [13]. These potential pharmacological properties of FAEE have been related to its increased lipophilicity compared to its acid precursor, a property that could enhance the accessibility to the intracellular medium by diffusion by the cell membrane [14].

The enhanced pharmacological properties of FAEE compared to FA is not an isolated phenomenon. Indeed, the esterification of phenolic acids has widely been used as an efficient approach to improve its efficacy in several experimental models. A significant example is gallic acid, since the antimutagenicity activity against activated 2-aminoanthracene (2AA)-induced SOS responses in *Salmonella typhimurium* is dependent on alkyl chain length of the gallic acid alkyl esters [15]. The antioxidant capacity, evaluated as inhibitors of AAPH-induced lyses of erythrocytes was eight-fold higher when propyl gallate was compared to gallic acid [16]. Gallates are also more effective than their acid precursors as inhibitors of TNF-α-induced NFκB activation in human embryonic kidney cells [17]. A similar tendency was observed to protocatechuic and caffeic acids. In this concern, findings in our laboratory have demonstrated that the heptyl ester of protocatechuic acid was ten-fold more efficient that the acid precursor as inhibitor of the production of superoxide in activated neutrophils [18]; and the butyl and heptyl esters of caffeic acid were four-fold more effective as bactericidal agent against *Helicobacter pylori* [19].

As well-known, exposure of the skin to ultraviolet B (UVB) radiation causes oxidative damage to skin, and ROS represent a pivotal role in this deleterious process [20]. Hence, compounds with antioxidant capacity are frequent components in sunscreen products [21]. Particularly with respect to FA, its presence in numerous cosmetic formulation and sunscreen products is a consequence of its antioxidant and photoprotective efficacy [8,22,23]. Regarding FAEE, the incorporation in O/W creams, at a concentration of 10%, gave a sun protection factor (SPF) similar to that of benzymidazilate, a filter permitted in the EU [24].

NADPH oxidases (Noxs), a group of multicomponent enzymes which catalyze the one-electron reduction of molecular oxygen, are found in a variety of cells, including phagocytes [25] and keratinocytes [26]. The activation of NADPH oxidase involves the migration of the cytosolic proteins to the membrane, enabling assembly of the enzyme complex, which release superoxide anion and, by an enzymatic cascade of reaction, to other ROS [25]. It has been demonstrated that UVA activates Nox1 to produce ROS that stimulate PGE2 synthesis, and that Nox1 may be an appropriate target for agents designed to block UVA-induced skin injury [27].

Considering the discovery that esterification of protocatechuic acid improved its efficacy as inhibitor of NADPH oxidase and the involvement of this multicomponent enzyme in the generation of ROS in skin, here we aimed to study and compare FA and FAEE regarding, antioxidant activity, inhibition of ROS released by leukocytes and their rheological characteristics in an emulsion based cream.

## 2. Results and Discussion

**Figure 1:**
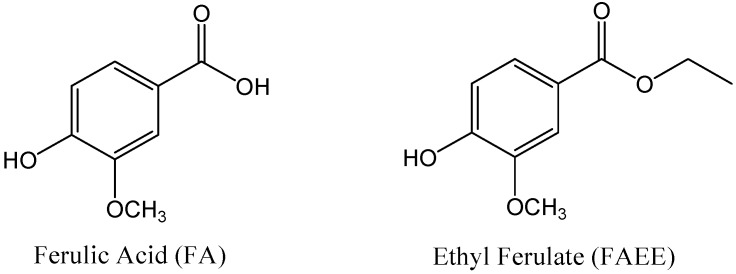
Molecular structure of ferulic acid (FA) and ethyl ferulate (FAEE).

FA and FAEE (Figure 1) have been widely used in cream for topical use. Among other factors, the application of these compounds is based in their antioxidant capacity [8,22,23,24,28]. Hence, our first objective was to compare their efficacies using a panel of *in vitro* antioxidant assays. From the data presented at Table 1, it can be observed that the esterification provoked a decrease in the capacity of FA as reducing agent toward the stable free radical DPPH. However, FAEE was still a better scavenger of DPPH compared to BHT, a widely used antioxidant for cosmetic preparations. We also used Trolox, a soluble form of vitamin E frequently used as a reference antioxidant. Trolox was the most efficient antioxidant in this experimental model.

**Figure 2:**
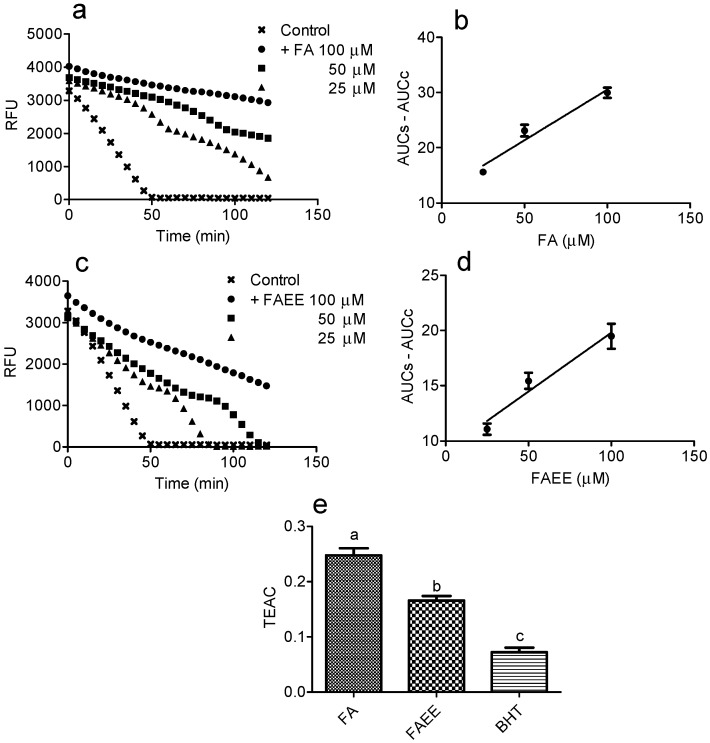
Scavenging activity against ROO•. (**a**,**c**) Kinetic profile of pyranine fluorescence bleaching by ROO• in the presence of absence of the tested compounds. (**b**,**d**) Linear relationship between the area under the curve and concentrations of FA and FAEE. From the liner regression curves the slopes were calculated. (**e**) Trolox equivalent antioxidant activity (TEAC = slope compound/slope trolox). The results are mean and SD of triplicate. Different letters denotes significant differences. One-Way Anova and Tukey’s Multiple Comparison Test (*p* < 0.05).

The next assay for comparison of anti-radical efficacy was based in the capacity of reduction of peroxyl radicals (ROO•). As well-established, ROO• are transient species in lipoperoxidation chain reactions, which are well-accepted as a pathway involved in the ROS-mediated cell damage [29]. Here, ROO• was generated by the thermolysis of the azo-compound AAPH, which decomposes at physiological temperature (37 °C) in aqueous solutions to generate an alkyl radical (R•). The R• reacts with molecular oxygen being converted to ROO• [30]. Figure 2a,c show the effect of FA and FAEE in the decay of pyranine fluorescence. The relationship between AUC and concentration of the tested compounds was the analytical parameter for the efficacy as ROO• scavenger (Figure 2b,d). The antioxidant efficacy was compared with Trolox, which was submitted to the same protocol and the Trolox equivalent antioxidant capacity (TEAC) calculated (Figure 2d). As can be observed, again FA was a more efficient antioxidant compared to FAEE and BHT.

**Figure 3:**
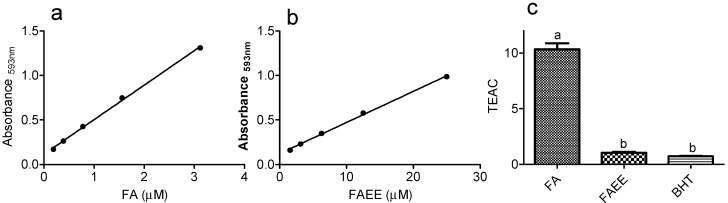
Ferric reducing capacity (FRAP assay). (**a**,**b**) Linear relationship between the concentration of the tested compounds and absorbance of the complex Fe(II)-TPTZ. From the liner regression curves the slopes were calculated. (**c**) Trolox equivalent antioxidant activity (TEAC = slope compound/slope trolox). The results are mean and SD of triplicate. Different letters denotes significant differences. One-Way Anova and Tukey’s Multiple Comparison Test (*p* < 0.05).

FA and FAEE were also compared by their capacity to reduce Fe(III) to Fe(II) using the FRAP assay (Figure 3). In this protocol the antioxidant capacity is measured by the formation of the complex Fe(II)-TPTZ, which absorbs at 593 nm [31]. Figure 3 shows the relationship between the concentration of the tested compounds and the absorbance of the Fe(II)-TPTZ complex. From the slopes, the TEAC was calculated as demonstrated in previous assays. How can be observed FA was again more efficient than FAEE.

**Table 1:** DPPH Scavenging Activity.

	IC_50_ (μM)
FA	23.5 ± 0.5
FAEE	59.7 ± 0.2
BHT	65.0 ± 0.5
Trolox	17.4 ± 0.3

The results are mean and SD of triplicates.

**Figure 4:**
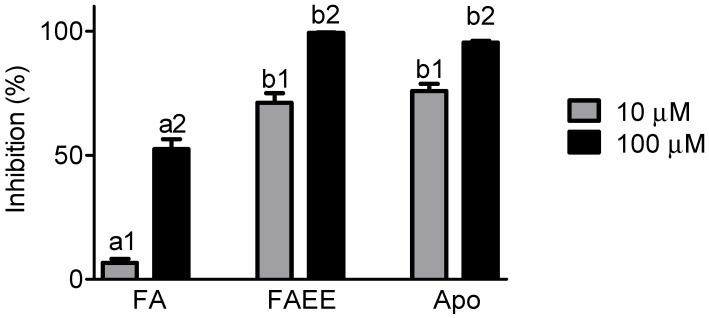
Inhibitory effect on ROS produced by stimulated neutrophils. The cells were stimulated by opsonized zymosan and the production of ROS evaluated by luminol-dependent chemiluminescence. The results are mean and SD of duplicates of three different experiments. Different letters denotes significant differences. One-Way Anova and Tukey’s Multiple Comparison Test (*p* < 0.05). The different concentrations of the tested compounds were treated separately in the statistical analysis (a_1_, b_1_ and c_1_ for 10 µM and a_2_, b_2_ and c_2_ for 100 µM.

As stated above, our main reason for studying the properties of FAEE in emulsions was its potential anti-inflammatory efficacy, as has been widely suggested [10,11,12,14]. Hence, we measured and compared FA and FAEE as inhibitors of the generation of ROS produced by stimulated neutrophils. For that, cells were isolated from peripheral blood and activated by opsonized zymosan. The release of non-specific ROS by the activated cells was evaluated by luminol-dependent chemiluminescence. From the results depicted in Figure 4, it can be seem that FAEE was significantly more potent than FA. Moreover, its efficacy was similar to apocynin, a compound widely used as NADPH oxidase inhibitor [32,33].

It must be emphasized that the luminol-dependent chemiluminescence assay is a technique that detects ROS like superoxide anion, hydrogen peroxide, hypochlorous acid, *etc.* Hence, in this experimental model, the inhibitory effect of a tested substance can be a consequence of direct scavenger action upon these ROS, by the inhibition of its generation, or both. For these reason, we also compared FA and FAEE using the chromogenic probe WST-1. This compound is a cell-impermeable and water soluble tetrazolium salt that reacts specifically with superoxide anion [34].

**Figure 5:**
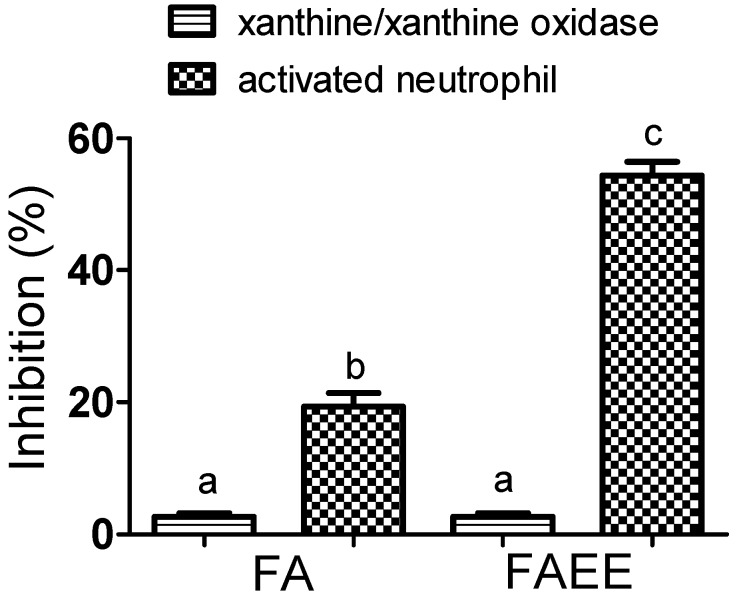
Inhibitory effect on superoxide anion produced by stimulated neutrophils and by xanthine/xanthine oxidase (X/XO). The production of superoxide was monitored by the reduction of WST-1. The tested compounds were used at 100 µM in both assays. The resultsare mean and SD of duplicates of three different experiments. Different letters denotes significant differences. One-Way Anova and Tukey’s Multiple Comparison Test (*p* < 0.05).

The data depicted in Figure 5 show that FAEE showed the same effect of apocynin and, again, it was more effective than FA. It is noteworthy that superoxide anion is the product of the activation of NADPH oxidase, hence this result could imply the inhibition of this enzyme complex or the scavenger of superoxide anion. To discriminate these effects we also tested the compounds in a cell-free system. As it can be observed, the effect of FA and FAEE using xanthine- xanthine oxidase as a source of superoxide, was much lower compared to the cell-system. Hence, we can conclude that FAEE is able to inhibit NADPH oxidase and this property could contribute to its anti-inflammatory activity.

As well-known, beyond the biological activity of its active substances, others factors are important for a topical formulation, such as the stability of the system, specially the stability of the active substances incorporated in the cream, the rheological behavior and the ability of the formulation in the release of the active substances. For that, FA and FAEE were incorporated in the base cream at 0.038% and 0.044% respectively. These values were chosen to reach 2 mM as final concentration, in which the biological effects assayed before should be guaranteed.

**Figure 6:**
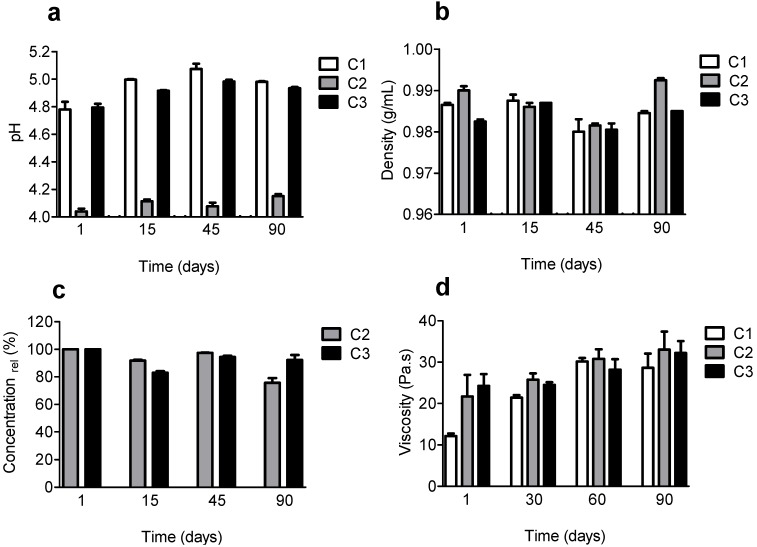
Stability of the creams. The creams were stored in transparent PVC packages at room temperature. After daylight exposure for up to 90 days (**a**) pH, (**b**) density, (**c**) concentration and (**d**) viscosity of the creams were evaluated. C1 (base cream), C2 (basecream plus FA) and C3 (base cream plus FAEE). The results are mean and SD of triplicates.

The creams were named C1 (base cream), C2 (base cream plus FA) and C3 (base cream plus FAEE). The formulations were submitted to the determination of organoleptic features (variations in appearance, phase separation, smell and color), pH, density, viscosity and degradation of the antioxidant components for 90 days at room temperature and daylight exposure. The organoleptic features were not modified during the 90 days of analysis (data not shown). For C1 and C3 the pH was around 4.8 and after 90 days only a slight increase was observed, that is not significant since they remained at a pH compatible with the skin, being suitable for topical use. As could be expected due to the acid character, the addition of FA (C2) decreased the pH of the emulsion to about 4.2 in relation to C1 and again this value was not changed significantly after 90 days (Figure 6a). The addition of FA and FAEE did not provoke a significant alteration in the density of the base cream and the obtained values did not change significantly after 90 days (Figure 6b). The presence of FA and FAEE provoked an increase in the viscosity of the base cream. However, after 15 days, no significant difference was observed among C1, C2 and C3. The viscosity of the formulations C1, C2 and C3 was only slightly increased during the 90 days (Figure 6c), probably due to water loss in formulations, which increases the viscosity due to the increase in the concentration of greasy components. FA and FAEE were also resistant to degradation. The result from Figure 6d show that the relative concentration of FA and FAEE was not changed significantly during the studied time interval. Altogether, these results are indicative that the presence of FA and FAEE did not cause any significant alteration in the physical properties of the base cream. These results allow concluding that in relation to the stability, both FA and FAEE were suitable active substances to produce a topical formulation, maintaining its stability when in contact with an emulsion-based formulation.

**Figure 7:**
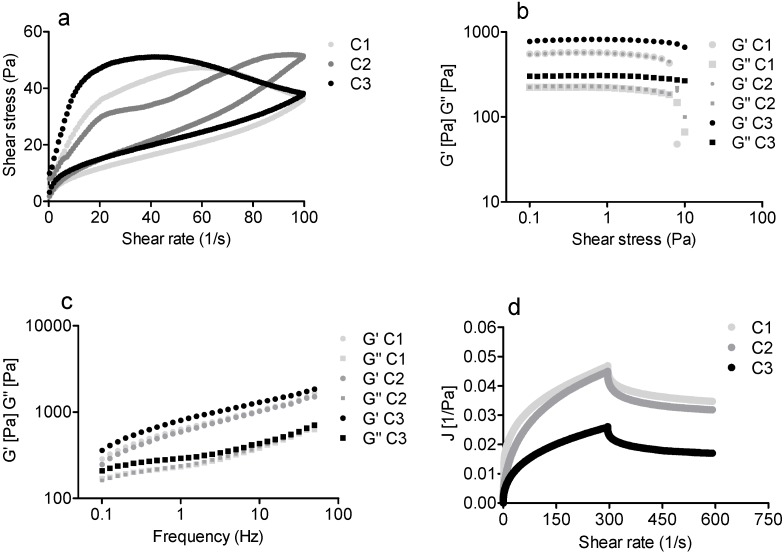
Rheological studies of the creams. (**a**) Flow curves, (**b**) stress sweep, (**c**) frequency sweep and (**d**) creep and recovery. **C1** (base cream), **C2** (base cream plus **FA**) and **C3** (base cream plus **FAEE**).

The formulations were submitted to rheological studies, aiming to verify their suitability to be used as topical products in relation mainly to their spreadability and viscosity. The first assay performed was the flow curves (Figure 7a). All the formulations were non-Newtonian and thixotropyc fluids. These features are suitable for formulations of topical use, indicating easy spreadability. Comparing the formulations, the hysteresis area of C1 and C2 were equivalent, but C3 had a higher hysteresis area.

Stress and frequency sweep tests were also performed, which were carried out to analyze the elastic modulus (G') and viscous modulus (G''). The results obtained with the stress sweep showed that the formulations could be submitted to a stress ranging from 0.1 to 10 Pa without significant structural alterations. Thus, 1 Pa was the shear stress chosen for the development of frequency sweeps and creep and recovery assay, ensuring that the formulation would not suffer any structure deformations during the assays. The results also show that compared to the base cream, the addition of FA and FAEE did not provoke significant alterations in these rheological properties (Figure 7b). The frequency sweep tests showed that G' was higher than G'' for all formulations (Figure 7c). Thus, it means that the formulations are more elastic than viscous, which could indicate a high stability of these systems, which is confirmed by the stability studies [35,36].

The viscoelastic features of the formulations were confirmed in the creep and recovery rheological assay, which also allows discriminating between the elastic and viscous responses (Figure 7d). The results also show the capacity of recovery which was of 19.19% for C1, 21.75% for C2 and 26.18% for C3. As can be observed, the presence of FAEE provoked an increase in the viscosity as was verified in the flow curves. These results suggest that formulation C3 is less susceptive to deformation, which will interfere with its spreadability.

**Figure 8:**
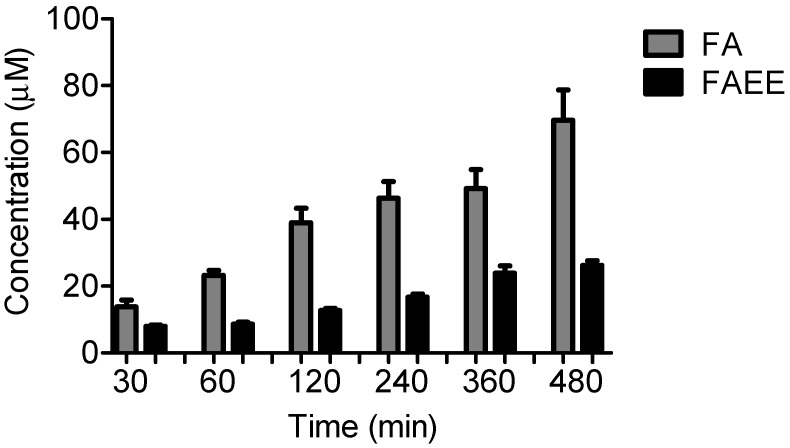
Release profile of creams containing FA and FAEE performed on Franz cells. Data represent the mean and SD of at least 2 different experiments conducted in triplicate.

Another important characteristic of C3 was the lower rate of release of the active principle compared to C2. Figure 8 shows that 80% of FA was released from C2 in eight hours, whereas C3 released only 25% of FAEE. This probably happened due to the higher hydrophobicity of FAEE, which could cause a higher association with the oily phase of the formulation.

## 3. Experimental

### 3.1. Chemicals

Ethyl ferulate, ferulic acid, (±)-6-hydroxy-2,5,7,8-tetramethylchromane-2-carboxylic acid (Trolox), 2,2'-azobis(2-amidinopropane) hydrochloride (AAPH), 2,2-diphenyl-1-picrylhydrazyl (DPPH), 8-hydroxypyrene-1,3,6-trisulfonic acid trisodium salt (pyranine), 2,4,6-Tri(2-pyridyl)-s-triazine (TPTZ), dimethyl sulfoxide (DMSO), Brij^®^ 35, tung oil, Histopaque^®^-1077, Histopaque^®^-1119, 3-(4,5-dimethylthiazol-2-yl)-2, 5-diphenyl tetrazolium bromide (MTT), phorbol 12-myristate 13-acetate (PMA), xanthine, xanthine oxidase and zymosan were purchased from Sigma-Aldrich Chemical Co. (St. Louis, MO, USA). 2-(4-Iodophenyl)-3-(4-nitrophenyl)-5-(2,4-disulfophenyl)-2H-tetrazolium monosodium salt (WST-1) was purchased from Santa Cruz Biotechnology (Santa Cruz, CA, USA). All reagents used for buffers and mobile phases were of analytical grade. Stock solutions of antioxidants for cellular and enzymatic studies were prepared in DMSO. Ultrapure Milli-Q water from Millipore (Belford, MA, USA) was used for the preparation of buffers and solutions. PMA solution was prepared in DMSO at a concentration of 16.0 M. Butylated hydroxytoluene (BHT), caprilyc capric triglyceride, ceteareth-20, cetearyl alcohol, cetyl palmitate, EDTA, octyl stearate, PHENOVA^®^ (phenoxyethanol, methylparaben, ethylparaben, propylparaben and butylparanen), and propylene glycol were purchased from Mapric Pharmacist Products Ltda (São Paulo, SP, Brazil).

### 3.2. Antioxidant Activity: DPPH Scavenging Assay

The relative antiradical potency of ferulic acid and its ester were compared by characterizing their capacity to reduce DPPH [37]. The tested compounds were incubated for 30 min with 100 µM DPPH in methyl alcohol in the dark. The scavenger activity was evaluated spectrophotometrically at 517 nm, using the absorbance of unreacted DPPH radical as a control. The scavenger activity was calculated as: [(absorbance of control − absorbance of sample)/(absorbance of control)] × 100.

### 3.3. Antioxidant Activity: Peroxyl Radical Scavenging Assay

These studies were performed as previously described with modifications [38]. The fluorescent compound pyranine (5 µM) was incubated with 20 mM AAPH in 10 mM phosphate buffered saline (PBS, pH 7.4) at 37 °C in the absence (control) or presence of the tested compounds in the wells of a microplate for 3 h. The final reaction volume was 300 µL. The fluorescence bleaching of the pyranine was monitored at 460/510 nm in a Spectramax M2 microplate reader (Molecular Devices, Sunnyvale, CA, USA). The curves (AUCsample—AUCcontrol) against the concentration of the tested compounds were raised and their slopes used as analytical parameter. Trolox was used as a reference antioxidant. The (slope_sample_/slope_Trolox_) ratio generated the Trolox equivalent antioxidant activity (TEAC), which we used to evaluate the relative antioxidant efficacy.

### 3.4. Ferric ion Reducing Antioxidant Power (FRAP) Assay

The FRAP reagent was prepared as follows: TPTZ (1 mL, 10 mM dissolved in 40 mM HCl), FeCl_3_ (1 mL, 20 mM dissolved in water) and sodium acetate buffer (10 mL, 300 mM, pH 3.6). The tested compounds at various concentrations (10 µL) were incubated with 290 µL of FRAP reagent for 30 min in the dark and room temperature [31]. The absorbance was measured at 593 nm using a mixture constituted of 10 µL PBS and 290 µL FRAP as a blank. The curves absorbances against the concentrations of the tested compounds were raised and their slopes used as analytical parameter. Trolox was used as a reference antioxidant. The ratio (slopes_ample_/slope_Trolox_) generated the Trolox equivalent antioxidant activity (TEAC), which we used for evaluate the relative antioxidant efficacy.

### 3.5. Isolation of Human Neutrophils and Peripheral Blood Mononuclear Cells

Blood samples were obtained from healthy volunteers. Experiments were performed in accordance with regulations of the Research Ethics Committee, Faculty of Pharmaceutical Sciences, Unesp, São Paulo, Brazil). Polymorphonuclear cells (PMN) were isolated by centrifugation on a Histopaque^®^-1077/1119 gradient at 700 × *g* for 30 min at room temperature. After isolation, the cells were resuspended in PBS supplemented with 1 mM calcium chloride, 0.5 mM magnesium chloride, and 1 mg/mL glucose (supplemented PBS) [39]. The cells were studied for cytotoxic effects of the tested substances using the trypan blue exclusion assay. At the higher concentration used (100 µM), the viability of the cells was >98% (data not shown).

### 3.6. ROS Production by Activated Leukocytes: Luminol-Dependent Chemiluminescence Assay

PMN (1 × 10^6^ cells/mL) were pre-incubated at 37 °C in supplemented PBS with the tested compounds for 15 min. Next, luminol (2.0 × 10^−5^ M) and opsonized zymosan (1.0 mg/mL) were added and the light emission was measured for 30 min at 37 °C using a plate luminometer (Centro Microplate Luminometer LB960, Berthold Technologies, Oak Ridge, TN, USA). The final assay volume was 250 μL. The integrated light emission was used as an analytical parameter for inhibition the ROS produced by the stimulated cells. The inhibitory potency was calculated using the light emission generated by the control, in which the zymosan-activated cells were incubated in the absence of the tested compounds [40].

### 3.7. Superoxide Anion Production by Activated Neutrophils

PMN (1.0 × 10^6^ cells/mL) were pre-incubated at 37 °C in supplemented PBS with the tested compounds for 10 min. Next, WST-1 (500 µM) and PMA (100 nM) were added and the reduction of WST-1 was measured spectrophotometrically at 450 nm for 30 min at 37 °C using a plate reader (Molecular Devices). The inhibitory potency was calculated using the absorbance of the control in which the PMA-activated cells were incubated in the absence of the tested compounds [18].

### 3.8. Superoxide Anion Radical Production by Xanthine/Xanthine Oxidase

The tested compounds (100 µM) were incubated at 37 °C in PBS with 500 µM WST-1 and 100 mM xanthine. The reactions were initiated by addition of 0.05 unit/mL xanthine oxidase and the reduction of WST-1 was measured spectrophotometrically at 450 nm for 15 min at 37 °C using a plate reader (SpectraMax M2, Molecular Devices) [18].

### 3.9. Preparation of Emulsions

The emulsions were prepared according to the usual technique. Oily and aqueous phase components were weighed separately. They were heated to 70 °C in water bath until solubilization and/or fusion of solid components. Aqueous phase was added to the oily phase under manual agitation until the temperature fell to room temperature. Oily phase: 6% cetearyl alcohol, 1% ceteareth-20, 1.5% cetyl palmitate, 1.5% octyl stearate and 1% caprilyc capric triglyceride. Aqueous phase: 3% propylene glycol, and water until 100 g. As preservatives 0.05% EDTA, 0.05% BHT, 0.2% for mixture of preservatives (phenoxyethanol, methylparaben, ethylparaben, propylparaben, butylparaben, isobutylparaben) were used. FA (0.038%) or FAEE (0.044%) were incorporated in the oily phase. The emulsions were named base cream (C1), base cream plus FA (C2) and base cream plus FAEE (C3).

### 3.10. Stability Studies

The creams were stored in transparent PVC packages at room temperature (24 ± 2 °C) and exposed to the daylight for up to 90 days. Samples were collected at the first day, 15, 45 and 90 days and evaluated for the organoleptic features, density, pH, chemical stability and viscosity [41]. For the chemical stability study, an analytical curve (concentration *versus* absorbance) of FA and FAEE solubilized in methanol were constructed using a spectrophotometer (Shimadzu, UV mini 1240, Kyoto, Japan) at 298 and 299 nm, respectively. Thus, periodically, samples of the emulsions exposed to the various stress conditions were collected, solubilized in methanol and determined the amount of FA and FAEE.

### 3.11. Rheological Studies

The rheological assays performed were: (a) flow curves, with shear rate varying from 0 to 100 s^−1^ during 120 s and then returning to 0 s^−1^ in 120 s; (b) stress sweep, with shear stress from 0.1 to 10 Pa and frequency at 1 Hz; (c) frequency sweep, with frequency from 0.1 to 50 Hz and shear stress of 1 Pa; (d) creep and recovery, with duration of 600 s, shear stress at 1 Pa and recovery to 0 Pa in 300 s. These tests were performed at 25 °C. The creams were evaluated using a rheometer (TA instruments, AR 2000 ex, New Castle, DE, USA) with a cone/plate sensor (1.59°, 40 mm) and the data analyzed by the Rheology Advantage Instruments Control Software [36].

### 3.12. Study of the Release Profile of FA and FAEE from the Creams

The evaluation of the release of FA and FAEE from the creams was performed as described [42]. Using Franz diffusion cells with 1.77 cm^2^ of diffusion area (Hanson Research, Microette, CA, USA), where a cellulose acetate membrane (Whatman^®^, 0.45 µM) was positioned between the donor compartment, containing the formulation and an acceptor compartment filled with phosphate buffer 0.1 M pH 7,4 and 5% DMSO. The temperature was kept at 37 ± 2 °C by a water jacket. The acceptor fluid was magnetically stirred (300 rpm) through the experiment. The time intervals for sample drawing were: 0.5, 1.0, 2.0, 4.0, 6.0 and 8.0 h. The relative concentrations of FA and FAEE were measured by their absorptions at 298 nm and 299 nm, respectively. The sink conditions were kept.

## 4. Conclusions

The scientific literature shows that FAEE has several potential beneficial health properties. Here, we have added one more data point to this panel of biological effects; its higher efficacy as an inhibitor of the generation of oxidants by activated leukocytes. As we have demonstrated, this effect was not related to its antiradical scavenging capacity. Indeed, FA was significantly more efficient as an antioxidant in the cell-free assays, but less effective in the cell-based assay. Specifically, our results suggest that FAEE acts as a NADPH oxidase inhibitor, and its potency was similar to that of apocynin, a widely used inhibitor of this enzymatic complex. When, incorporated in a topical pharmaceutical formulation, FAEE caused some rheological alterations, such as increased viscosity and lower susceptibility to deformation compared to the base cream or cream with FA. The lower spreadability can be useful for formulations that are developed for application on restrict areas of the skin, such as around the eyes. Hence, the use of FA or FAEE in creams should be decided in conformity with their intended applications. FA when the purpose is a better antiradical effect or FAEE if anti-inflammatory properties are desired.
